# *Vachellia farnesiana* Pods or a Polyphenolic Extract Derived from Them Exert Immunomodulatory, Metabolic, Renoprotective, and Prebiotic Effects in Mice Fed a High-Fat Diet

**DOI:** 10.3390/ijms24097984

**Published:** 2023-04-28

**Authors:** Claudia Delgadillo-Puga, Dulce R. Sánchez-Castillo, Yonatan Y. Cariño-Cervantes, Ivan Torre-Villalvazo, Claudia Tovar-Palacio, Sarai Vásquez-Reyes, Janette Furuzawa-Carballeda, Joshua Ayork Acevedo-Carabantes, María del Rayo Camacho-Corona, Jorge Luis Guzmán-Mar, Luis Cisneros-Zevallos, Armando R. Tovar, Rosa Rebollar-Vega, Georgina Hernández-Montes, Alfredo Ulloa-Aguirre, Berenice Palacios-Gonzalez, Lilia G. Noriega

**Affiliations:** 1 Departamento de Nutrición Animal Dr. Fernando Pérez-Gil Romo, Instituto Nacional de Ciencias Médicas y Nutrición Salvador Zubirán (INCMNSZ), Mexico City 14080, Mexico; yair221c@gmail.com; 2 Facultad de Química, Universidad Nacional Autónoma de México, Mexico City 04510, Mexico; dulce.rose9726@gmail.com; 3 Departamento de Fisiología de la Nutrición, Instituto Nacional de Ciencias Médicas y Nutrición Salvador Zubirán (INCMNSZ), Mexico City 14080, Mexico; ivan.torrev@incmnsz.mx (I.T.-V.); ogawa.kaoru69@gmail.com (S.V.-R.); stuff.joshua@gmail.com (J.A.A.-C.); armando.tovarp@incmnsz.mx (A.R.T.); 4 Dirección de Nutrición, Instituto Nacional de Ciencias Médicas y Nutrición Salvador Zubirán (INCMNSZ), Mexico City 14080, Mexico; claudia.tovarp@incmnsz.mx; 5 Departamento de Cirugía Experimental, Instituto Nacional de Ciencias Médicas y Nutrición Salvador Zubirán (INCMNSZ), Mexico City 14080, Mexico; jfuruzawa@gmail.com; 6 Facultad de Ciencias Químicas, Universidad Autónoma de Nuevo León (UANL), Av. Universidad s/n, Ciudad Universitaria, San Nicolás de Los Garza 66455, Mexico; maria.camachocn@uanl.edu.mx (M.d.R.C.-C.); jorge.guzmanmr@uanl.edu.mx (J.L.G.-M.); 7 Department of Horticultural Sciences, Texas A&M University, College Station, TX 77843, USA; lcisnero@tamu.edu; 8 Red de Apoyo a la Investigación, Universidad Nacional de Autónoma de México, Instituto Nacional de Ciencias Médicas y Nutrición Salvador Zubirán, Mexico City 14080, Mexicoyinna@cic.unam.mx (G.H.-M.); aulloaa@unam.mx (A.U.-A.); 9 Unidad de Vinculación Científica Facultad de Medicina, Instituto Nacional de Medicina Genómica 14, (INMEGEN), Mexico City 16080, Mexico; bpalacios@inmegen.gob.mx

**Keywords:** *Vachellia farnesiana* pods, high-fat diet, insulin resistance, insulin secretion, anti-obesity, naringenin, methyl gallate, bioactive compounds, mitochondrial function, 16S rDNA

## Abstract

Obesity causes systemic inflammation, hepatic and renal damage, as well as gut microbiota dysbiosis. Alternative vegetable sources rich in polyphenols are known to prevent or delay the progression of metabolic abnormalities during obesity. *Vachellia farnesiana* (VF) is a potent source of polyphenols with antioxidant and anti-inflammatory activities with potential anti-obesity effects. We performed an in vivo preventive or an interventional experimental study in mice and in vitro experiments with different cell types. In the preventive study, male C57BL/6 mice were fed with a Control diet, a high-fat diet, or a high-fat diet containing either 0.1% methyl gallate, 10% powdered VFP, or 0.5%, 1%, or 2% of a polyphenolic extract (PE) derived from VFP (*Vachellia farnesiana* pods) for 14 weeks. In the intervention study, two groups of mice were fed for 14 weeks with a high-fat diet and then one switched to a high-fat diet with 10% powdered VFP for ten additional weeks. In the in vitro studies, we evaluated the effect of a VFPE (*Vachellia farnesiana* polyphenolic extract) on glucose-stimulated insulin secretion in INS-1E cells or of naringenin or methyl gallate on mitochondrial activity in primary hepatocytes and C2C12 myotubes. VFP or a VFPE increased whole-body energy expenditure and mitochondrial activity in skeletal muscle; prevented insulin resistance, hepatic steatosis, and kidney damage; exerted immunomodulatory effects; and reshaped fecal gut microbiota composition in mice fed a high-fat diet. VFPE decreased insulin secretion in INS-1E cells, and its isolated compounds naringenin and methyl gallate increased mitochondrial activity in primary hepatocytes and C2C12 myotubes. In conclusion VFP or a VFPE prevented systemic inflammation, insulin resistance, and hepatic and renal damage in mice fed a high-fat diet associated with increased energy expenditure, improved mitochondrial function, and reduction in insulin secretion.

## 1. Introduction

The chronic intake of hyperenergetic diets causes an increase in fat mass leading to overweight and obesity. Obesity is a major risk factor for several metabolic chronic diseases, including type 2 diabetes, metabolic fatty liver disease, atherosclerosis, neurodegenerative disorders, chronic kidney disease, and cardiovascular diseases such as heart disease and stroke, which are the leading causes of death worldwide [1]. Excessive fat mass induces lipotoxic cellular damage leading to the dysfunction of different tissues and organs, such as white and brown adipose tissue, pancreas, liver, skeletal muscle, and kidney, among others [2]. The altered physiological processes include mitochondrial dysfunction, impaired intracellular energy-sensing mechanisms, aberrant activation of metabolic nuclear receptors, and gut microbiota dysbiosis. Altogether, these alterations induce oxidative stress, cellular necrosis, and activation of immune-inflammatory pathways, leading to hyperinsulinemia, insulin resistance and glucose intolerance, metabolic inflexibility, impaired substrate oxidation, and energy expenditure [3,4,5,6]. Given the severity of the perils that obesity posits, developing new and diverse strategies to prevent and treat obesity and its metabolic consequences is of great interest. This type of concern ranges from including healthy eating habits to synthetic drugs [7]. In recent years, the interest in anti-obesity medicinal plants has increased significantly in several countries due to their affordability, availability, and phytochemical content [8]. Polyphenols represent the second largest group of plant phytochemical content. Chemically, polyphenols are defined as a group of natural compounds characterized for containing one or several phenolic rings. Plants synthesize a highly diverse number of polyphenols and encompass more than 8000 polyphenolic substances [9], which exert beneficial metabolic effects and, therefore, have begun to be used to prevent or treat obesity and its metabolic disorders. At the cellular and molecular levels, these secondary metabolites exert antioxidant, anti-inflammatory, and intracellular signaling activities, preventing metabolic abnormalities and cellular damage in several tissues and organs, including a decrease in lipotoxicity in skeletal muscle, as well as an improvement in liver dysfunction [8]. More recently, we have shown in rodent models of diet-induced obesity that consumption of goat milk, soy protein, or black bean (*Phaseolus vulgaris*) extracts that contain a significant amount of polyphenols exert anti-obesity, anti-inflammatory, and anti-diabetic activities. The molecular mechanisms of these beneficial effects include increased mitochondrial biogenesis in skeletal muscle, increased UCP-1 expression in brown adipose tissue, reduced hepatic lipogenesis, prevention of gut microbiota dysbiosis, increased energy expenditure, reduced inflammation, and increased whole-body insulin sensitivity [10,11,12,13,14].

The search for new non-conventional plants with beneficial polyphenols capable of modulating multiple metabolic targets without side effects has been a great challenge for the scientific community. In this sense, our research group has studied the beneficial effects of one of these non-conventional vegetable sources of polyphenols, *Vachellia farnesiana* pods (VFP). *Vachellia farnesiana* (L.) Wight and Arn. (synonym *Acacia farnesiana* (L.) Willd.) is a woody Mimosoideae legume common in tropical and subtropical regions, also known as mimosa bush, sweet acacia, cassie, and Huizache [15]. VFP are green at the beginning and turn brown when ripe. They are small, 5 to 8 cm long on average, and reach maturity during the dry season in Mexico when sources of forage are limited. Thus, VFP is an excellent alternative to forage for husbandry animal production. Additionally, their consumption transfers bioactive compounds and pro-health properties to animals and their products [16]. We have isolated and characterized the constituents of the fruits of *Vachellia farnesiana* [17] and demonstrated the anti-inflammatory and antioxidant effect of a phenolic extract of VFP in gerbils and mice [18,19]; we have also observed the antimicrobial action of methyl gallate isolated from *Vachellia farnesiana* [20] and the antimicrobial activity of VFP against *Mycobacterium tuberculosis* [21]. Finally, we have demonstrated that feeding a high-fat diet to mice along with milk from goats fed a conventional diet supplemented with 30% VFP increased energy expenditure, augmented oxidative fibers in skeletal muscle, and reduced inflammatory markers. Thus, we wonder whether those positive effects could be recapitulated by the direct consumption of VFP or a polyphenolic extract derived from them and whether isolated compounds from VFP, such as methyl gallate and naringenin, exert direct actions on mitochondrial function and insulin secretion in vitro.

Thus, the present study aimed to evaluate the effect of VFP or three concentrations of a polyphenolic extract derived from VFP (VFPE) in vivo on weight gain, liver steatosis, insulin resistance, immune cells profile, renal damage, and the microbiota composition of mice fed a high-fat diet. Additionally, we evaluated the in vitro effect of VFPE on the modulation of insulin secretion on INS1E cells and its isolated compounds, methyl gallate and naringenin, on the mitochondrial function of primary hepatocytes and C2C12 myotubes.

## 2. Results

### 2.1. A Phenolic Extract of Vachellia farnesiana Pods (VFPE) Contains Significant Amounts of the Bioactive Compounds Methyl Gallate and Naringenin

**Figure 1:**
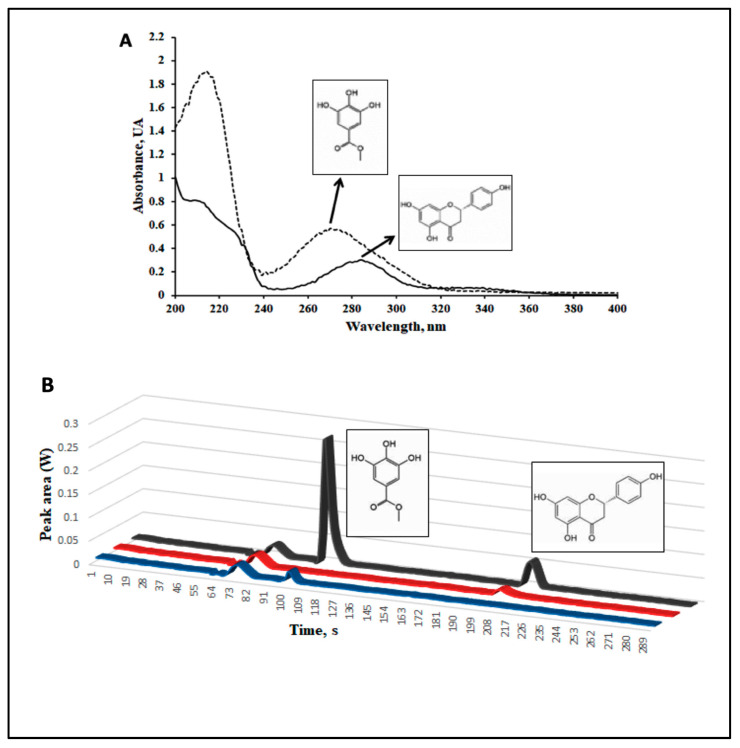
Quantification of polyphenols in a *Vachellia farnesiana* extract (**A**) UV-Vis absorption spectrum for methyl gallate (MG, 270 nm) and naringenin (NA, 283 nm). (**B**) Chromatographic separation of a standard solution showing peaks for methyl gallate (MG), blue line, and naringenin (NA), red line; the retention time (tR) was 102 and 209 s, respectively. The peaks found in the VFE are shown by the black line.

Many of the beneficial effects of edible plants in the body are related to their content of phenolic compounds since they can exert antioxidant, immunomodulatory, and genomic effects, the latter through modulation of nuclear receptor activity and the chromatin remodeling machinery [22]. To determine the abundance of specific phenolic compounds in VFP, we quantified methyl gallate (MG) and naringenin (NA) content in VFPE. One gram of lyophilized VFPE (raw methanol:water 80:20 extract) was passed through column chromatography on silica gel (20 g) and eluted with a gradient of hexane/EtOAc. Each fraction was analyzed on TLC, and the fractions rich in MG were combined in pooled fractions. Likewise, fractions rich in NA were combined with other pooled fractions. The pooled fractions were analyzed to quantify MG and NA using high-performance liquid chromatography with a charge-coupled device detector. The absorption spectra of each compound and the chromatogram are shown in Figure 1A,B.

Quantification was performed by external calibration, based on peak areas, against the standard of MG and NA. Regarding calibration, the slope obtained for routine calibration was compared with the slope of the standard addition method. The results indicated no matrix effects for evaluated samples, so external calibration was used throughout this study. The calibration curves of the HPLC-CCD system were constructed for mixed standard concentrations of 10 to 80 mg/L with a correlation coefficient (r) in the range of 0.9990–0.9991. The pooled fractions for MG (1.0 mg) and NA (4.0 mg) were dissolved in 10 mL of methanol and analyzed by HPLC-CCD. The concentrations obtained for each pooled fraction were 62.85 ± 0.98 mg/g for MG and 26.30 ± 1.13 mg/g for NA according to the calibration curve (Supplementary Materials, Figure S1).

### 2.2. VFP or a VFPE Prevents Excessive Weight Gain, Fat Mass Accumulation, and Lean Mass Loss in Mice Fed a High-Fat Diet

**Figure 2:**
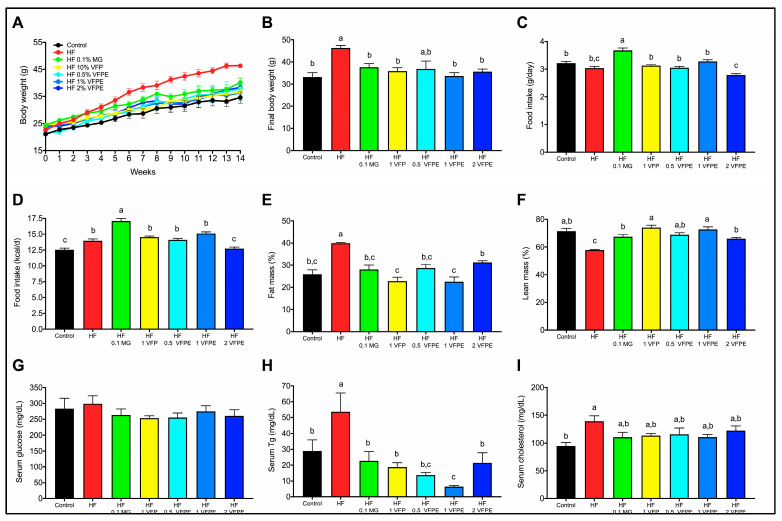
Body weight gain, food and energy intake, body composition, and biochemical parameters of mice fed HF diets containing VFP or VFPE. (**A**) Body weight gain throughout the study, (**B**) final body weight, (**C**) daily food intake in grams, (**D**) daily energy intake in kcal, (**E**) percentage of fat mass, (**F**) percentage of lean mass, (**G**) serum glucose, (**H**) triglycerides, and (**I**) cholesterol of mice fed with Control diet (Control), high-fat diet (HF), or HF diet containing 0.1% methyl gallate (HF 0.1 MG), 10% *Vachellia farnesiana* pods (HF 1 VFP), 0.5% (HF 0.5 VFPE), 1% (HF 1 VFPE), or 2% (HF 2 VFPE) of a phenolic extract derived from *Vachellia farnesiana* pods. Results are presented as the mean ± S.E.M., *n* = 6 mice per group, and analyzed by one-way ANOVA followed by Tukey multiple comparison post hoc tests. The differences were considered statistically significant at *p* < 0.05. Mean values with different lowercase letters show statistical differences between each other.

Chronic consumption of high-energy diets causes excessive fat mass accretion and induces several metabolic derangements, such as reduced lean mass and altered lipid profile. To evaluate the effect of VFP or VFPE on body composition and biochemical parameters, we fed mice with a high-fat (HF) diet or an HF diet containing 10% VFP or 0.5%, 1%, or 2% VFPE for 14 weeks. The body weight gain of mice fed the HF diet was higher than that of mice fed the Control diet (Control) during the study period (Figure 2A,B). The increased body weight in these mice was due to augmented body fat and reduced body lean mass. Interestingly, mice fed with HF diets containing VFP or VFPE presented a body weight gain similar to Control mice but also a significant reduction in fat mass (Figure 2E) and an increase in lean mass (Figure 2F) concerning HF mice. The beneficial effects of VFP and VFPE in body composition were not due to a reduction in food intake since animals fed VFP or VFPE presented similar food and energy intake for Control and HF groups throughout the study (Figure 2C,D) or even higher, especially in mice fed MG. To evaluate the effect of VFP and VFPE in serum biochemical parameters, we determined circulating glucose, triglycerides (Tg), and total cholesterol. We did not find differences in fasting glucose concentration in either group (Figure 2G). However, serum Tg was significantly lower in mice fed HF containing VFP and VFPE than in those fed HF (Figure 2H). The serum cholesterol of mice fed VFP and VFPE was similar to that of HF but also similar to that of the Control (Figure 2I). These results show that VFP and VFPE intake prevents the increase in body weight gain, fat mass, and circulating Tg and lean mass loss caused by chronic consumption of an HF diet.

### 2.3. The Consumption of VFP or VFPE Improves Glucose and Insulin Tolerance, Prevents Pancreatic Islets Hypertrophy in Mice Fed a High-Fat Diet, and Notably, the VFPE Decreases Insulin Secretion in a Dose-Dependent Manner in INS-1E Cells

**Figure 3:**
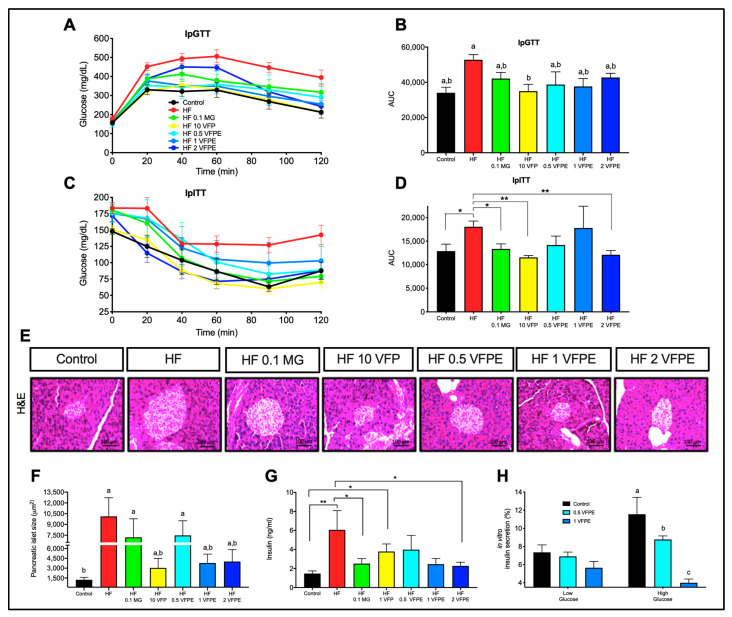
Glucose tolerance, pancreatic islet size of mice fed HF diets containing VFP and VFPE, and in vitro insulin secretion. (**A**) Glucose concentrations during intraperitoneal glucose tolerance test (ipGTT), (**B**) ipGTT area under the curve (AUC), (**C**) glucose concentrations during intraperitoneal insulin tolerance test (ipITT), (**D**) ipITT AUC, (**E**) representative hematoxylin and eosin stained pancreatic islets, (**F**) islet size quantification, (**G**) insulin concentration in serum of mice fed with Control diet (Control), high-fat diet (HF), or HF diet containing 0.1% methyl gallate (HF 0.1 MG), 10% *Vachellia farnesiana* pods (HF 10 VFP), 0.5% (HF 0.5 VFPE), 1% (HF 1 VFPE), or 2% (HF 2 VFPE) of a phenolic extract derived from *Vachellia farnesiana* pods. Results are presented as the mean ± S.E.M., *n* = 6 mice per group, and analyzed by one-way ANOVA followed by Tukey multiple comparison post hoc tests. The differences were considered statistically significant at *p* < 0.05. Mean values with different lowercase letters show statistical differences between each other. An unpaired *t*-test was employed to analyze the comparison of treatments * statistically significant at *p* < 0.05; ** at *p* < 0.001. (**H**) VFPE effect on insulin secretion in INS-1E cells. Digital photographs were taken from each section at 20×.

To evaluate the effect of VFP or VFPE on glucose and insulin tolerance of mice fed an HF diet, we performed an ipGTT and an ipITT. Mice fed with HF showed a significant (*p* < 0.05) higher area under the curve (AUC) when compared to Control mice during the ipGTT (Figure 3A,B) and the ipITT (Figure 3C,D). Interestingly, mice fed either dose of the VFPE had an AUC intermediate between the Control and the HF mice in both curves. Notably, the most substantial effect was observed with VFP, which had the same AUC as the Control diet and was significantly lower than the HF mice during the ipGTT (Figure 3B). Interestingly, the effect of VFP on glucose tolerance was associated with potentially higher insulin sensitivity since the VFP group also had the same AUC during the ipITT as the Control (Figure 3D). In line with this, mice fed with HF had higher pancreatic islets sizes than Control (Figure 3E,F). In contrast, mice fed with either VFP or VFPE had smaller pancreatic islet sizes despite consuming an HF diet and reduced fasting insulin concentration (Figure 3G). These results indicate that VFP and VFPE increase whole-body insulin sensitivity and glucose tolerance, which is reflected in a lower need for the pancreas to secrete insulin, preventing pancreatic islet hypertrophy. This feature is commonly associated with hyperinsulinemia in mice fed HF diets [23]. We hypothesize that in addition to peripheral actions in insulin-sensitive tissues, the VFPE may exert direct effects on pancreatic beta cells, modulating insulin secretion. Thus, to evaluate whether the VFPE could modulate insulin secretion in beta cells, we performed an in vitro experiment using rat insulinoma INS-1E cells incubated with 5 or 10 mg/mL of the VFPE in a low or high glucose culture medium. We found that the VFPE did not affect insulin secretion at low glucose concentrations. In contrast, at high glucose concentrations, VFPE produced a significant and dose-dependent decrease in insulin secretion compared to Control (Figure 3H). Altogether, these results indicate that VFP and VFPE attenuate whole-body glucose intolerance, insulin resistance, and VFPE, specifically decreasing excessive insulin secretion from the pancreas, preventing hyperinsulinemia and pancreatic islet hypertrophy in mice fed an HF diet.

### 2.4. The Consumption of VFP or VFPE Prevents the Decrease in Energy Expenditure Associated with a High-Fat Diet without Altering Substrate Utilization

**Figure 4:**
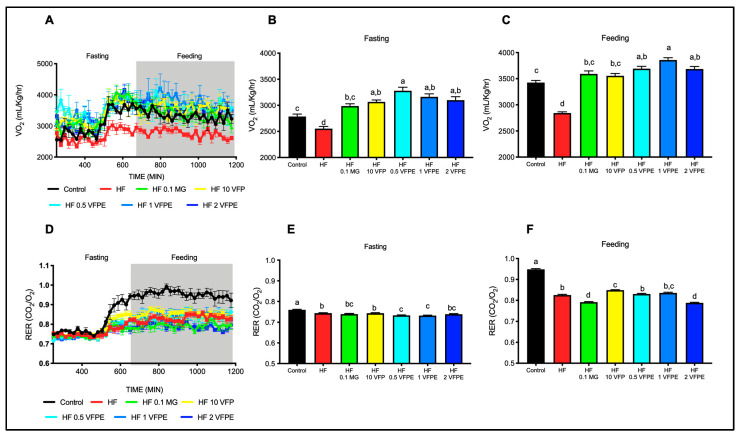
Whole-body energy expenditure and substrate oxidation of mice fed HF diets containing VFP and VFPE. (**A**) Oxygen consumption (VO_2_) during fasting and feeding periods was determined by indirect calorimetry analysis; clear and shaded zones indicate fasting and feeding periods, respectively. (**B**) Average oxygen consumption during fasting, (**C**) average oxygen consumption during feeding, (**D**) respiratory exchange ratio (RER) and average RER during (**E**) fasting and (**F**) feeding periods. Indirect calorimetry was performed in mice fed a Control diet (Control), high-fat diet (HF), or HF diet containing 0.1% methyl gallate (HF 0.1 MG), 10% *Vachellia farnesiana* pods (HF 10 VFP), 0.5% (HF 0.5 VFPE), 1% (HF 1 VFPE), or 2% (HF 2 VFPE) of a phenolic extract of *Vachellia farnesiana* pods. Results are presented as the mean ± S.E.M., *n* = 6 mice per group, and analyzed by one-way ANOVA followed by Tukey multiple comparison post hoc tests. The differences were considered statistically significant at *p* < 0.05. Mean values with different lowercase letters show statistical differences between each other.

The observation that mice fed VFP had lower body weight and improved glucose tolerance suggests that VFP intake can improve whole-body energy expenditure and substrate utilization. Thus, we measured oxygen consumption by indirect calorimetry using a CLAMS system. As expected, HF mice had lower average oxygen consumption than control mice in both fasting and feeding conditions (Figure 4A,B). Interestingly, VFP and VFPE intake at either dose showed a significantly higher oxygen consumption than HF and even higher than Control mice, especially at 1% of VFPE in fed conditions (Figure 4C). However, when we evaluated the effect of VFP or VFPE on substrate utilization by calculating the respiratory exchange ratio (RER), control mice had an RER of 0.75 during fasting conditions (Figure 4D,E), which increased to 9.5 during the fed state, showing the ability to change from fatty acids to glucose utilization when alternate substrates become available during the fasting-to-feeding transition. Conversely, the RER of HF mice remained close to 0.8 during the feeding period (Figure 4F), indicating the inability to change substrate utilization. Interestingly, mice fed with VFP presented a significantly higher RER than HF mice during feeding, indicating improved flexibility to switch substrates for oxidation. On the other hand, mice fed with the VFPE had lower RER, similar to what we have previously observed in mice fed with goat milk [10], which indicates a higher utilization of fatty acids as energy substrate even during the feeding period. These results indicate that VFP and VFPE intake increases energy expenditure and metabolic flexibility in mice fed an HF diet. These findings suggest an improvement in mitochondrial activity in metabolic tissues such as muscle or liver.

### 2.5. VFP or VFPE Increases Mitochondrial Abundance in the Skeletal Muscle of Mice Fed with an HFD, and VFPE Augments Mitochondrial Oxidative Metabolism in C2C12 Myotubes

**Figure 5:**
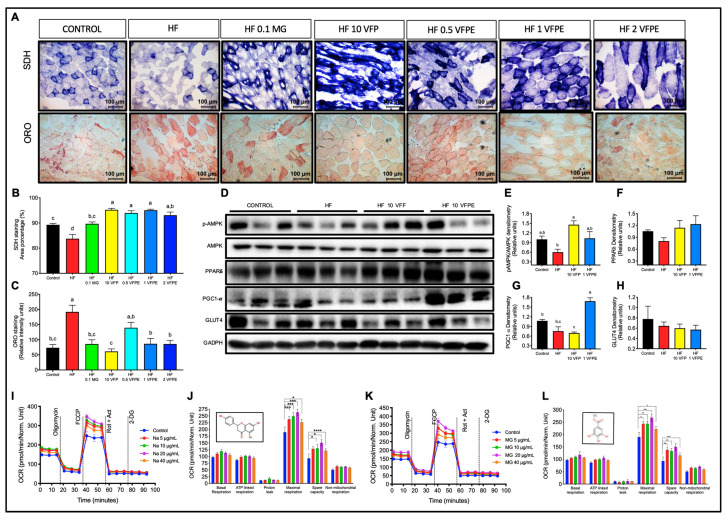
Skeletal muscle oxidative metabolism of mice fed HF diets containing VFP and VFPE. (**A**) Oil red O relative staining intensity, (**B**) Succinate dehydrogenase-nitro blue tetrazolium (SDH) staining, (**C**) oil red O and SDH staining in muscle tissue. (**D**) Immunoblot of p-AMPK, AMPK, PPARδ, PGC1-α, GLUT-4, and GADPH of mice fed with Control diet (Control), high-fat diet (HF), or HF diet containing 0.1% methyl gallate (HF 0.1 MG), 10% *Vachellia farnesiana* pods (HF 10 VFP), 0.5% (HF 0.5 VFPE), 1% (HF 1 VFPE), or 2% (HF 2 VFPE) of a phenolic extract obtained from *Vachellia farnesiana* pods. Relative abundance of (**E**) phospho-AMPK and AMPK, (**F**) PPARδ, (**G**) PGC1-α, and (**H**) GLUT-4 in muscle. Results are presented as the mean ± S.E.M., *n* = 6 mice per group, and analyzed by one-way ANOVA followed by Tukey multiple comparison post hoc tests. The differences were considered statistically significant at *p* < 0.05. Mean values with different lowercase letters show statistical differences between each other. Oxygen consumption rate (OCR) and the calculated mitochondrial parameters of C2C12 myotubes treated with naringenin (**I**,**J**) or methyl gallate (**K**,**L**) myotubes. Each bar represents the mean ± SEM (*n* = 3). * *p* < 0.05 and **, ***, **** *p* < 0.001 significantly different. Digital photographs were taken from each section at 20×.

To assess whether the augmented oxygen consumption observed in mice fed VFP or VFPE was related to an increase in skeletal muscle oxidative metabolism, we evaluated skeletal muscle mitochondrial activity through succinate dehydrogenase (SDH) and lipid content by ORO staining. We found a significant increase in SDH-positive fibers in mice fed VFP or VFPE with respect to those fed HF (Figure 5A,B). Interestingly, the increase in mitochondrial activity was associated with a reduction in lipid accumulation in myofibers. The relative intensity of ORO staining was significantly lower in mice fed with an HF diet that incorporates VFP or VFPE than those fed only the HF diet (*p* < 0.05) (Figure 5A,C). Mitochondrial biogenesis and activity in skeletal muscle are transcriptionally regulated by the peroxisome proliferator-activated receptor delta (PPARδ) and the PPAR gamma coactivator-1 alpha (PGC1-α) [24] and post-transcriptionally by the activity of the AMP-activated protein kinase (AMPK) [25]. Both PPARδ and AMPK can be activated by plant phenolic compounds, promoting many responses, such as glucose uptake and mitochondrial biogenesis, leading to the remodeling of muscle fiber type composition to a metabolically more oxidative and less glycolytic one. Interestingly, AMPK activity was higher in mice fed with VFP or VFPE than those fed the HF diet (Figure 5D,E). Similarly, the protein abundance of PPARδ and PGC1-α in skeletal muscle was significantly upregulated in the VFPE group concerning the HF group (Figure 5D–G). However, we did not find differences in the glucose transporter GLUT4 protein abundance between groups fed VF concerning HF (Figure 5D,H). These results indicate that the increased oxygen consumption and metabolic flexibility observed in mice fed VFP or VFPE is associated with an increased oxidative metabolism in skeletal muscle.

To evaluate whether the increase in oxidative fibers observed in mice fed with VFP or VFPE may be due to its polyphenol content, we performed a mitochondrial stress test in C2C12 myotubes treated with 5, 10, 20, or 40 μM of NA or MG for 18 h. As observed in Figure 5I–L, NA, and MG increased maximal mitochondrial respiration and the reserve respiratory capacity in a dose-dependent manner without altering other mitochondrial parameters such as basal and ATP-linked respiration, proton leak, or even non-mitochondrial respiration. Altogether, these results demonstrate that consumption of VFP or VFPE increases mitochondrial activity and oxidative metabolism in skeletal muscle that could be, in part, mediated by its phenolic content, which includes NA and MG.

### 2.6. VFP or VFPE Reduces Hepatic Lipid Content in Mice Associated with Increased Mitochondrial Activity in Primary Hepatocytes Exposed to VFPE

**Figure 6:**
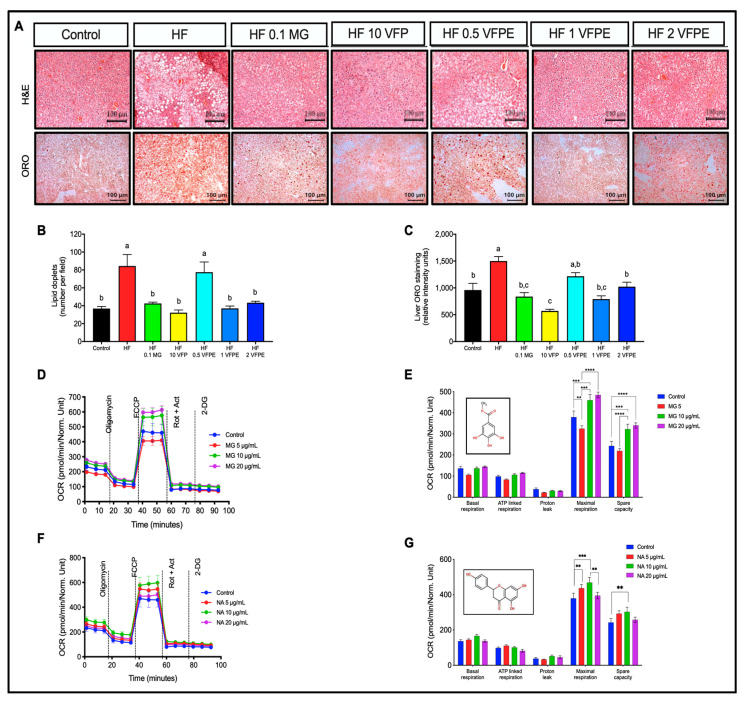
Hepatic tissue morphology in mice fed HF diets containing VFP and VFPE. (**A**) Digital photographs of H&E and oil red O staining of mice fed with Control diet (Control), high-fat diet (HF), or HF diet containing 0.1% methyl gallate (HF 0.1 MG), 10% *Vachellia farnesiana* pods (HF 10 VFP), 0.5% (HF 0.5 VFPE), 1% (HF 1 VFPE), or 2% (HF 2 VFPE) of a phenolic extract obtained from *Vachellia farnesiana* pods. (**B**) Lipid droplet area and (**C**) oil red O staining relative intensity of digital photographs in A. Results are presented as the mean ± S.E.M., *n* = 6 mice per group, and analyzed by one-way ANOVA followed by Tukey multiple comparison post hoc tests. The differences were considered statistically significant at *p* < 0.05. Mean values with different lowercase letters show statistical differences between each other. Oxygen consumption rate (OCR) and calculated mitochondrial parameters of primary hepatocytes treated with methyl gallate (MG) (**D**,**E**) or naringenin (NA) (**F**,**G**). Each bar represents the mean ± SEM (*n* = 3). ** *p* < 0.05 and ***, **** *p* < 0.001 significantly different. Digital photographs were taken from each section at 20×.

Chronic energy-dense diets such as HF diets lead to hepatic lipid accumulation that can progress to steatohepatitis and hepatic cirrhosis [26]. To determine the effect of VFP or VFPE on hepatic lipid content, we measured hepatic lipid droplet areas in H&E-stained sections and lipid accumulation in ORO-stained slides. As observed in Figure 6A–C, the percentage of lipid droplet areas and ORO-positive vacuoles were lower in the liver of mice fed VFP or VFPE in a dose-dependent manner with respect to those fed HF (*p* < 0.05). To evaluate whether the decrease in hepatic lipid content observed in mice fed VFP or the VFPE was associated with an improvement in mitochondrial function that could be attributed to the content of NA or MG, we performed a mitochondrial stress test in primary hepatocytes treated with 5, 10, or 20 μM of MG or NA for 18 h. Interestingly, MG increased maximal respiration and the reserve respiratory capacity at 10 or 20 μM (Figure 6D,E) without alteration of other mitochondrial parameters such as basal and ATP-linked respiration, proton leak, or even non-mitochondrial respiration. In addition, NA increased maximal respiration and reserve respiratory capacity at 5 and 10 μM without modifying the rest of the parameters (Figure 6F,G). Altogether, these results demonstrate that consumption of VFP or a VFPE decreases lipid content in the liver, probably attributed to an increase in mitochondrial activity in hepatocytes induced by the MG or NA present in the VFPE.

### 2.7. VFP or VFPE Consumption Prevents Visceral and Subcutaneous Adipocyte Hypertrophy Associated with Increased Thermogenic Activity in Brown Adipose Tissue of Mice Fed a High-Fat Diet

**Figure 7:**
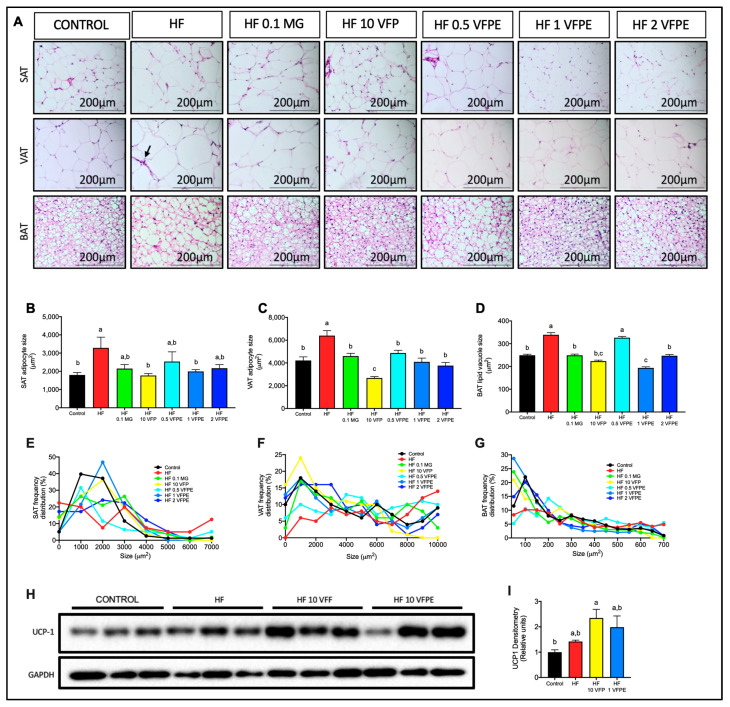
Subcutaneous (SAT), visceral (VAT), and brown (BAT) adipose tissue morphology and UCP1 expression. (**A**) Hematoxylin and eosin staining of SAT, VAT, and BAT from mice fed with Control diet (Control), high-fat diet (HF), or HF diet containing 0.1% methyl gallate (HF 0.1 MG), 10% *Vachellia farnesiana pods* (HF 10 VFP), 0.5% (HF 0.5 VFPE), 1% (HF 1 VFPE), or 2% (HF 2 VFPE) of a phenolic extract obtained from *Vachellia farnesiana* pods; (**B**) SAT, (**C**) VAT, and (**D**) BAT adipocyte size; (**E**) SAT, (**F**) VAT, and (**G**) BAT adipocyte size frequency distribution; (**H**) UCP-1 immunoblot from BAT of mice fed with the indicated treatments; (**I**) UCP-1 densitometric analysis. Results are presented as the mean ± S.E.M., *n* = 6 mice per group, and analyzed by one-way ANOVA followed by Tukey multiple comparison post hoc tests. The differences were considered statistically significant at *p* < 0.05. Mean values with different lowercase letters show statistical differences. Arrow mark aggregated macrophages around dead adipocytes. Digital photographs were taken from each section at 20×.

White adipose tissue (WAT) (visceral (VAT) and subcutaneous (SAT)) and brown adipose tissue (BAT) are the two main types of adipose tissue present in mammals; the former is responsible for energy storage via the synthesis and accumulation of triglycerides, while the latter regulates energy expenditure and body temperature through thermogenesis, i.e., the generation of heat through substrate oxidation in the mitochondria uncoupled from ATP production via UCP-1. However, in response to high-energy diets, WAT becomes dysfunctional, reducing its proliferative capacity and leading to adipocyte hypertrophy. Furthermore, chronic intake of HF diets also induces BAT dysfunction, reducing its capacity to oxidize stored lipids and dissipating energy as heat [27]. To evaluate if the consumption of VFP or a VFPE prevents the hypertrophy of SAT, VAT, or BAT, we measured adipocyte areas in H&E-stained sections. The adipocyte size in SAT and VAT were smaller in mice fed with VFP or VFPE than in HF-fed mice and were similar to Control (Figure 7A–C). The frequency distribution of adipocyte size showed that mice fed with VFP or VFPE had a higher number of small adipocytes ranging from 1000 to 10,000 μm^2^. In contrast, those fed HF had bigger adipocytes, ranging from 10,000 to 30,000 μm^2^ (Figure 7E,F). We observed numerous dead adipocytes in the VAT of mice fed HF diet (Figure 7A arrow), as identified by the characteristic crown-like structure surrounded by macrophages. Interestingly, VAT from mice fed with VFP or VFPE did not exhibit signs of macrophage recruitment or crown-like structures.

Interestingly, BAT adipocytes were also smaller in mice fed VFP or VFPE than in those fed HF, indicating increased lipid oxidation (Figure 7D,G). To evaluate if the reduced adipocyte size in BAT is associated with increased UCP-1 protein content, we evaluated UCP-1 in BAT by Western blot. As observed in Figure 7H,I, VFP or VFPE-treated mice exhibited a higher abundance of the browning marker UCP-1 in BAT than Control or HF-fed mice. These results indicate that VFPE can prevent WAT dysfunction and preserve BAT thermogenesis, which suggests that lipid oxidation and energy expenditure will be favored even on an HF diet.

### 2.8. VFP or VFPE Exert Reno-Protective Effects on Mice Fed a High-Fat Diet

**Figure 8:**
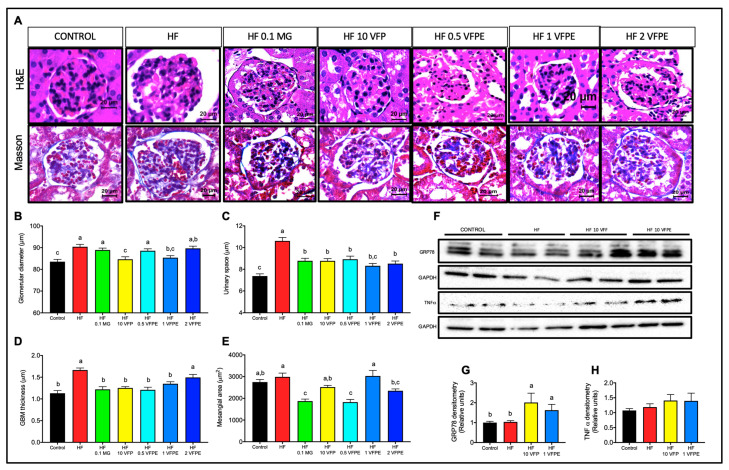
Kidney morphology and stress markers. (**A**) Hematoxylin and eosin (H&E) and Masson staining, (**B**) glomerular area, (**C**) urinary space size, (**D**) glomerular basement membrane (GBM) thickness, (**E**) mesangial area size, immunoblot of (**F**) GRP78 and TNF-α protein abundance, densitometric analysis of (**G**) GRP78, and (**H**) TNF-α protein abundance in kidneys of mice fed with Control diet (Control), high-fat diet (HF), or HF diet containing 0.1% methyl gallate (HF 0.1 MG), 10% *Vachellia farnesiana* pods (HF 10 VFP), 0.5% (HF 0.5 VFPE), 1% (HF 1 VFPE), or 2% (HF 2 VFPE) of a phenolic extract obtained from *Vachellia farnesiana* pods. Results are presented as the mean ± S.E.M., *n* = 6 mice per group, and analyzed by one-way ANOVA followed by Tukey multiple comparison post hoc tests. The differences were considered statistically significant at *p* < 0.05. Mean values with different lowercase letters show statistical differences between each other. Digital photographs were taken from each section at 20×.

High-fat diets induce several biochemical and structural abnormalities in the kidneys, leading to a decline in glomerular filtration rate, glomerulosclerosis, and tubulointerstitial fibrosis [28]. In the present study, we observed that an HF diet significantly increases glomerular size, indicating that obesity induced not only metabolic alterations but also altered the glomerular structure of the kidneys. Interestingly, when VFP or 1% of VFPE was added to the HF diet, glomerular size decreased to a size similar to the Control diet (Figure 8A,B). Histopathological evaluation of renal microscopic images indicates that the HF-diet induced alterations in Bowman’s space (Figure 8C) and increased the glomerular basement membrane (GBM) area (Figure 8D), along with the mesangial area (Figure 8E). Interestingly, in mice exposed to MG, VFF, or VFPE at different concentrations, the glomeruli area, GBM, and mesangial area were similar to the Control group. These results indicate that VFP or VFPE exert renoprotective activities in mice fed an HF-diet.

Moreover, most dietary polyphenols entering the bloodstream are eliminated from the body via the kidneys, which may increase the glomerular filtration rate when polyphenols are consumed in high quantities, inducing cellular stress [29,30]. To evaluate renal cellular stress, we measured the protein abundance of GPR78, an unfolded protein response (UPR) marker, and the pro-inflammatory cytokine TNF alpha. Figure 8F,G shows an increase in GRP78 protein content in the kidneys of mice fed with VFP or VFPE. Increased cellular energy demand triggers the UPR, a physiological response to several stressors to restore cellular homeostasis. The increase in GRP78 protein abundance is a marker of adaptive UPR in the kidney [31]. However, maladaptive UPR leads to inflammation and cell death when cellular homeostasis is not achieved. Importantly, TNF alpha protein abundance was not significantly different between groups (Figure 8F,H), indicating that the diet supplemented with VFP or VFPE does not induce cellular damage in the kidney.

### 2.9. VFP or VFPE Increases Circulating Anti-Inflammatory Immune Cells

**Figure 9:**
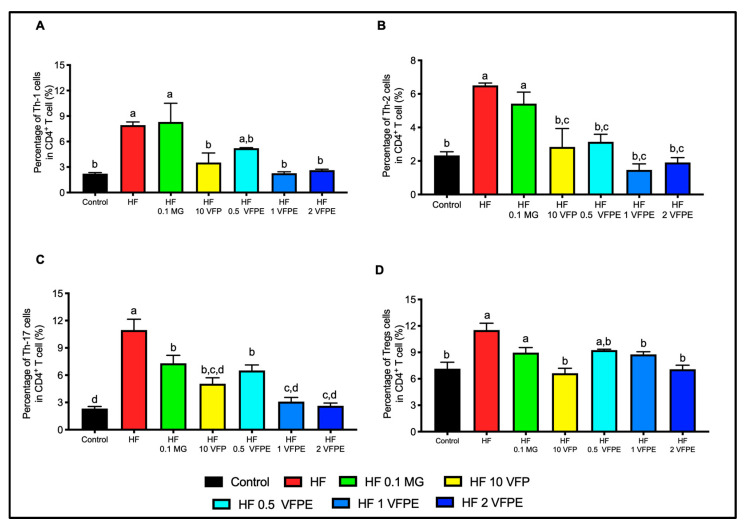
Frequency changes of splenic (**A**) Th-1, (**B**) Th-2, (**C**) Th-17, and (**D**) Treg cells in mice fed with Control diet (Control), high-fat diet (HF), or HF diet containing 0.1% methyl gallate (HF 0.1 MG), 10% *Vachellia farnesiana* pods (HF 10 VFP), 0.5% (HF 0.5 VFPE), 1% (HF 1 VFPE), or 2% (HF 2 VFPE) of a phenolic extract from *Vachellia farnesiana* pods. Results are presented as the mean ± S.E.M., *n* = 6 mice per group, and analyzed by one-way ANOVA followed by Tukey multiple comparison post hoc tests. The differences were considered statistically significant at *p* < 0.05. Mean values with different lowercase letters show statistical differences between each other.

The chronic intake of high-energy diets induces cellular damage in every tissue and cell type. This damage arises from mitochondrial dysfunction, oxidative stress, endoplasmic reticulum stress, protein nitrosylation, DNA fragmentation, and lipid peroxidation, among other insults. Damaged cells undergo necrotic cell death, releasing damage-associated molecular patterns (DAMPs), activating sterile inflammation, and recruiting proinflammatory immune cells [32]. As expected, in mice fed the HF diet, we found an increased percentage of CD4+ effector T cells (Th17, Th1, and Th2 by 3-, 5-, and 6-fold, respectively, compared with the Control (Figure 9A–C). An increase in Tregs cells by 1.5-fold was also observed in mice HF-fed compared to the Control group, which can be interpreted as a compensatory mechanism to resolve chronic systemic inflammation (Figure 9D). Interestingly, mice fed with VFP or VFPE showed a dose-dependent effect on the downregulation of CD4+ effector T subsets and Tregs. These results indicate that VFPE can prevent systemic inflammation and pro-inflammatory lymphocyte recruitment, leading to resolution and restoring immune homeostasis.

### 2.10. Effect of VFP or VFPE on Bacterial Taxonomy, Relative Abundance at Phylum and Genus Level, Alpha and Beta Diversity, and Linear Discriminant Analysis (LDA) Score of the 16S rRNA Sequencing of Feces in Mice Fed an HF-Diet

**Figure 10:**
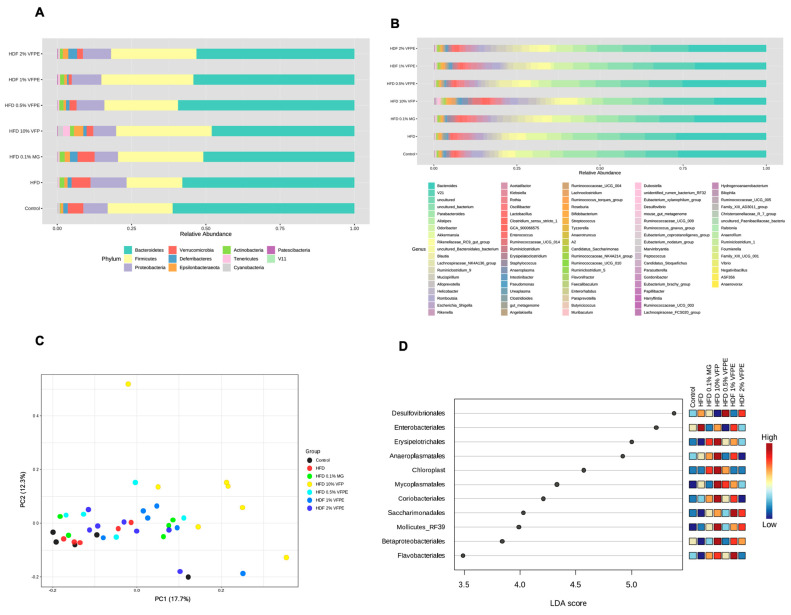
Bacterial taxonomy, relative abundance (%) at phylum (**A**) and genus level (**B**), (**C**) linear discriminant analysis (LDA) score and beta diversity (**D**) of the 16S rRNA sequencing of feces in mice fed with Control diet (Control), high-fat diet (HF), or HF diet containing 0.1% methyl gallate (HF 0.1 MG), 10% *Vachellia farnesiana* pods (HF 10 VFP), 0.5% (HF 0.5 VFPE), 1% (HF 1 VFPE), or 2% (HF 2 VFPE) of a phenolic extract from *Vachellia farnesiana* pods.

Mounting evidence has established the important role of intestinal microbiota composition in the metabolic and immune functions of the host, as a deleterious effect of high-fat diets is the alteration of these microbial communities [6]. We have previously demonstrated that saponins from *Agave salmiana*, another alternative vegetable source, can restore eubiosis of microbial communities in obese mice [14]. Thus, to evaluate if VFP or VFPE can modulate fecal gut bacterial composition, we performed fecal gut microbiota taxonomic classification by analyzing microbiome sequence data in the QIIME 2 microbiome bioinformatics platform. Figure 10A,B show the relative abundance (%) at the phylum and genus level of fecal bacterial communities of mice fed with HF diet or an HF diet containing VFP or VFPE. We then analyzed the indexes of Chao1 and Shannon to compare species richness (*p*-value: 0.58641; (ANOVA) F-value: 0.78703) and species diversity among groups (*p*-value: 0.24059; (ANOVA) F-value: 1.4092). However, we did not observe differences for any group. Interestingly, the beta diversity analysis (Figure 10D) showed a significant effect among groups where the VFP was different (*p* < 0.001) concerning the rest of the groups. Figure 10C shows the study groups’ linear discriminant analysis (LDA) effect size. A high LDA score indicates a more significant contribution of the taxa to the group separation. We observed a high difference among groups (*p* < 0.01). Surprisingly, the mice fed VFP showed a significant decrease in the abundance of *Desulfovibrionaceae*. Another relevant result is the reduction in the relative abundance of *Enterobacteriales* bacteria order in the feces of mice fed VFP or VFPE with respect to those fed only with HF. Interestingly, the incorporation of VFPE (0.5%, 1%, and 2%) reduced the relative abundance of the Gram-positive bacteria *Erysipelotrichales*, *Chloroplast, Mollicutes,* and *Mycoplasmatales* genus of the group of Firmicutes with respect to the rest of the groups (Figure 10D). These results indicate that VFP or VFPE can act as a prebiotic source that prevents the reduction in microbial communities’ diversity, preventing dysbiosis and immune disequilibrium.

### 2.11. Dietary Intervention with VFP Decreases Body Weight, Pancreatic Islet Size, and Adipocyte Hypertrophy in Obese Mice

**Figure 11:**
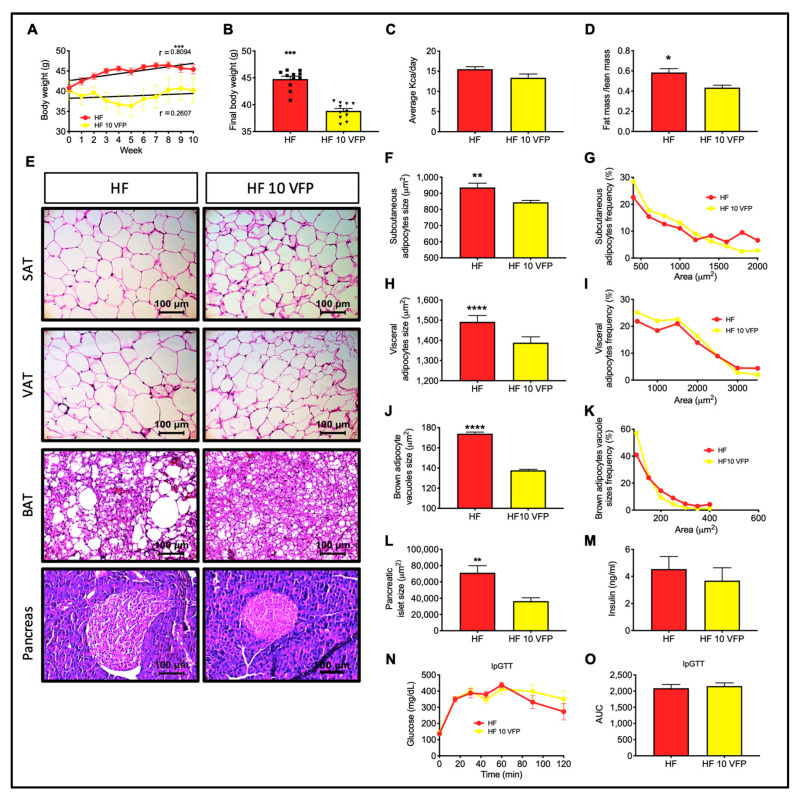
Therapeutic potential of *Vachellia farnesiana* pods in obese mice. (**A**) Body weight of obese mice after intervention with a high-fat diet with 10% *Vachellia farnesiana* fruits (HF 10 VFP) for ten weeks, (**B**) final body weight after the intervention, (**C**) average kcal consumed per mouse per day, and (**D**) body fat/lean mass ratio at the end of intervention. (**E**) Hematoxylin and eosin stained subcutaneous, visceral, brown adipocytes and pancreatic islets of mice fed HF or HF 10 VFP. (**F**) Subcutaneous adipocytes mean size, (**G**) subcutaneous adipocytes frequency distribution, (**H**) visceral adipocytes mean size, (**I**) visceral adipocytes frequency distribution, (**J**) brown adipocytes lipid droplets mean size, (**K**) brown adipocytes frequency distribution, and (**L**) pancreatic islets size quantification in mice fed HF or HF 10 VFP. (**M**) Fasting serum insulin concentration, (**N**) glucose tolerance, and (**O**) blood glucose AUC of obese mice after intervention with HF 10 VFP. Results are presented as the mean ± S.E.M., *n* = 6 mice per group, and analyzed by unpaired two-tailed *t*-test. The differences were considered statistically significant at * *p* < 0.05 snd **, ***, **** *p* < 0.001. Digital photographs were taken from each section at 20×.

As VFP intake prevented excessive body weight gain, fat mass accretion, visceral and subcutaneous adipocyte hypertrophy, and conversely, increased glucose and insulin tolerance, we tested the therapeutic potential of VFP intake through an intervention study in diet-induced obese mice that were also glucose intolerant. Consumption of VFP for ten weeks significantly reduced the body weight (Figure 11A,B) of obese mice, and this difference was not due to reduced energy intake (Figure 11C). The lower body weight of mice fed VFP was caused by a reduction in the fat/lean mass ratio, indicating a reduction in adipose tissue mass (Figure 11D). Accordingly, VFP intake reverted subcutaneous and visceral adipocyte hypertrophy (Figure 11E–I) and restored thermogenic BAT morphology, denoted by a significant reduction in lipid vacuoles (Figure 11E,J,K). VFP also reverted pancreatic islet hypertrophy (Figure 11E,L). However, fasting insulin or glucose tolerance was similar between groups (Figure 11M–O). These results demonstrate that VFP intake can partially improve mice’s metabolic alterations induced by chronic high-fat diet intake.

## 3. Discussion

### 3.1. HPLC Quantification of Methyl Gallate (MG) and Naringenin (NA) in Vachellia Farnesiana Extract

Various polyphenols have been shown to exert health benefits through antioxidant, immunomodulatory, and prebiotic activity and act as ligands for metabolic nuclear receptors. For this reason, edible plants containing significant amounts of phenolic compounds are now denominated functional foods. Polyphenols are part of the natural compounds in foods or non-conventional vegetable sources, such as VF. To gain insight into the phenolic content in VFP, we aimed to determine the phenolic profile in a VFPE by HPLC. However, it was not achieved due to the great variety of complex compounds that it contains. We decided then to fractionate the extract by column chromatography on silica gel and a gradient of Hexane/EtOAc. The fractions obtained were then analyzed by thin-layer chromatography. Fractions containing MG and NA were pooled separately [33]. Then, the fractions enriched with each secondary metabolite were analyzed separately in HLC to determine the amount of MG and NA in each fraction using a calibration curve for both MG and NA. We found a considerable amount of MG (62.85 mg/g) and NA (26.30 m/g) in the extract (see Supplementary Materials).

### 3.2. VFPE Prevents Excessive Body Weight Gain in Mice Fed a High-Fat Diet by Increasing Whole-Body Oxygen Consumption and Skeletal Muscle Mitochondrial Activity

The chronic intake of hypercaloric diets leads to the development of metabolic inflexibility, that is, the impaired metabolic transition between fatty acids and glucose during the daily fasting and feeding cycles. High-energy diets also impair whole-body oxidative metabolism, which is reflected in reduced oxygen consumption and energy expenditure. This leads to impaired glucose and lipid metabolism and an increase in body weight due to the accumulation of fat mass. These metabolic alterations are primarily the result of mitochondrial dysfunction in energy-demanding tissues such as skeletal muscle and the liver [34]. In humans, skeletal muscle composes up to 40% of the adult body weight and accounts for 18% of the resting metabolic rate. Moreover, higher mitochondrial oxidative capacity in skeletal muscle is associated with an increased resting metabolic rate [35]. We found that VFP or VFPE increased whole-body metabolic flexibility and oxygen consumption in mice fed an HF diet, which resulted in lower body weight, lesser fat mass, and augmented glucose tolerance and insulin sensitivity concerning those fed HF. Accordingly, our results showed a significant increase in skeletal muscle mitochondrial activity in mice fed VFP or VFPE with respect to HF. The same results were observed in cultured myocytes administered with MG or NA. The mechanisms involved in the significant effects of VF polyphenols in skeletal muscle physiology can be due to the modulation of nuclear receptor activity. In recent years, knowledge about the interaction of nutrients with metabolic nuclear receptors, such as the peroxisome proliferator-activated receptors (PPARs), has increased considerably. Mitochondrial biogenesis and function, energy expenditure, glucose, and lipid metabolism are under the control of the PPAR family of nuclear receptors. These nuclear receptors sense and respond to endogenous ligands, such as free fatty acids and their derivatives, and exogenous ligands, such as plant phenolics, to regulate genes involved at almost all levels of lipid metabolism, including lipid import/export, synthesis, storage, and oxidation [28]. In skeletal muscle, PPARδ is the most abundant PPAR isoform and is mainly expressed in the type I muscle fiber phenotype, characterized by elevated mitochondrial density and oxidative metabolism [36,37]. Our results suggest that polyphenol compounds present in VFP or VFPE act as ligands for PPARδ in skeletal muscle, increasing mitochondrial content, increasing whole-body oxygen consumption and metabolic flexibility, and preventing glucose intolerance, body weight gain, and excess fat mass accretion.

### 3.3. VFP or VFPE Reduces Plasma Triglycerides and Hepatic Lipid Content

Chronic intake of hypercaloric diets induces hepatic lipid accumulation, leading to hepatic steatosis and dyslipidemia [38]. The cause of the altered hepatic lipid metabolism during the consumption of HF diets is primarily an aberrant activation of nuclear receptors such as PPARα, PPARγ, and liver-X-receptors (LXRs), augmenting fatty acids synthesis, triglyceride esterification, and lipoprotein secretion [39,40]. Incorporating polyphenol-rich extracts in regular diets is one important strategy that has been developed to modulate the activities of these receptors during obesity [14,41]. Citrus flavonoids such as naringenin and naringin are known ligands of metabolic nuclear receptors [42], producing effects similar to those exerted by fenofibrate [43]. A recent HPLC-Q-TOF-MS analysis by our group has identified these compounds in methanolic and aqueous extracts of VFP [21]. Our in vivo results show that VF polyphenols reduce hepatic and circulating lipids. Accordingly, our in vitro experiments in cultured primary hepatocytes showed an increase in maximal respiration and reserve respiratory capacity in the presence of NA or MG. Therefore, the reduction of hepatic and circulating lipids observed in mice fed VFP or VFPE can be ascribed to mitochondrial activity stimulation by the citrus flavonoid in VF.

### 3.4. Intake of VFP or VFPE Prevents Subcutaneous and Visceral Adipocyte Hypertrophy and Preserves Brown Adipose Tissue Thermogenic Morphology

Chronic positive energy balance leads to excessive energy storage as triglycerides in white adipose tissues, leading to adipose tissue expansion. However, the pro-inflammatory and pro-oxidant environment that develops during obesity reduces adipocyte proliferation and vascularization, leading to adipocyte hypertrophy and hypoxia [44]. Interestingly, an HF diet containing VFP or VFPE prevented subcutaneous and visceral adipocyte hypertrophy and hypoxia, as observed by the absence of macrophage-surrounded dead adipocytes. The probable mechanisms of preservation of adipocyte functionality by VFPE include antioxidant and anti-inflammatory activities or modulation of the adipogenic nuclear receptor PPARƔ. Concerning BAT, the VF-derived polyphenols preserved their thermogenic morphology, characterized by multiple small vacuoles and numerous mitochondria, along with an increased UCP-1 protein abundance. These results indicate that VFPE preserves white and brown adipose tissue functionality, preventing excessive lipid spillover to peripheral organs and increasing thermogenic glucose and fatty acid oxidation. These beneficial effects can be in part responsible for the reduced lipid accumulation in skeletal muscle and liver and increased whole-body glucose tolerance observed in mice fed VFP or VFPE.

### 3.5. Intake of VFP or VFPE Is Not Nephrotoxic

Polyphenols are known as renoprotective compounds due to their antioxidant and anti-inflammatory properties. However, all phenolic compounds entering the body are eliminated primarily by the kidney and, to a lesser extent, by the bile. Thus, it can increase glomerular filtration, particularly when consumed in high quantities [29,30]. We found that VFP or VFPE prevented glomerular damage induced by the HF diet without exerting energy or osmotic stress, as evaluated by the abundance of the endoplasmic reticulum stress marker GRP78 and the cytokine TNFα. These results indicate that polyphenols from VFP or VFPE exert renoprotective activities in mice fed an HF diet without nephrotoxicity.

### 3.6. VFP or VFPE Restored Immune Homeostasis in Mice Fed with a High-Fat Diet, with a Dose-Dependent Effect on the Downregulation of CD4+ Effector T Subsets and Tregs Cells

We determined the circulating lymphocyte profile to determine if VFPE exerts immunomodulatory activities. The CD4+ T helper cells differentiate into T helper (Th)1, Th2, Th9, Th17, and Th22, depending on the cytokine environment [45]. The IL-17A-expressing CD4 T cells are a key mediator of autoimmune diseases. Abadja et al. [46] and Dardalhon et al. [47] mentioned that under physiological and pathological conditions, Th17 cells induce B cell proliferation and differentiation into immunoglobulin-secreting cells and produce a wide range of pro-inflammatory cytokines such as IL-17A, IL-17F, IL-21 and IL-22, IL-1β, IL-6, IL-8, TNF-α, IL-23, G-CSF, GM-CSF, and chemokines (CXCL1, CXCL2, CCL2, CXCL5, and CCL20). This endorses the diapedesis of neutrophils and monocytes. The increased infiltration of Th17 cells is associated with enlarged IL-21 concentrations and activation-induced cytidine deaminase (AID) expression [48]. AID is the key enzyme that Controls Ig class switching and somatic hypermutation, suggesting that IL-17 indirectly promotes lymphoid process, considered beneficial in reparative processes concerning ongoing injury. Nonetheless, sustained activity may result in the continuous production of various wound-healing growth factors, ultimately becoming a pathological process leading to fibrosis [40,41,42,43,44,45,46,47,48,49,50,51,52].

According to the functional characteristics of effector CD4 T cells, it is clear that an increase in the proportion of these subsets favors the triggering, development, and maintenance of chronic inflammatory diseases [53]. In contrast, regulatory cells constitute 5% of neogenesis and support the development of humoral immune responses contributing to antibody-mediated response [54]. Interferon-gamma (IFN-γ) synthesized by Th1 cells promotes cell-mediated immunity, activates mononuclear cells, and regulates various immune and inflammatory responses. Specifically, IFN-γ promotes immune modulation and has anticancer and antimicrobial activity. It inhibits type I collagen synthesis and produces chemokines and their receptors [47]. IL-4-expressing Th2 cells are a pleiotropic cytokine involved in regulating the immune response. They inhibit the synthesis of pro-inflammatory cytokines, promote Th2 cell differentiation, and inhibit autoimmune disease mediated by Th1 cells. It is a B cell stimulator factor and is a downregulator of apoptosis. IL-4 influences cytokine during fibrosis [55]. IL-13, a Th2-related cytokine, is a key mediator in fibroproliferative disorders. It acts through the regulation of the type I collagen gene and plays an essential role in the polarization of macrophages/dendritic cells to the M2a phenotype [49,50]. In this way, M2a macrophages are 15% of peripheral and tissue CD4^+^ T cells. Tregs modulate the natural course of protective immune responses maintaining immune tolerance to limit tissue damage, allergy, inflammation, cancer, and autoimmunity. Treg-mediated suppressive activity includes the synthesis of granzyme B, adenosine, CAMP, and perforins; the production of IL-10 and IL-35; the depletion of IL-2; the expression of suppressor molecules such as CTLA-4, GITR, LAG-3, IL-19, and TGF-β1; and antigen-presenting cells (APCs) decrease functions that otherwise promote energy or apoptosis of effector T cells. Thus, the Tregs play a key role in metabolic and genetic regulation, tissue repair, and homeostasis [56]. It was shown that dietary polyphenols are immunomodulators reducing Th1 differentiation and numbers of Th17 and Th9 cells, upregulating the number of Tregs, and maintaining the balance between Th17/Treg, attenuating inflammation [45]. In this study, we demonstrate that VFP or its VFPE can restore the balance of CD4^+^ effector T cells/regulatory T cells, allowing homeostasis at the peripheral level in mice fed an HF diet.

### 3.7. Bacterial Taxonomy, Abundance Relative (%) at Phylum and Genus Level of the 16S rRNA Sequencing of Feces in Mice Fed with HF Diet or VFP or VFPE

The gut microbiota are now accepted as one of the main environmental factors involved in the development of obesity. Previous reports have confirmed that the gut microbiota play a significant role in fat mass deposition and energy homeostasis disbalance [57]. Using the linear discriminant analysis (LDA), we evaluated the effect size among the study groups to evaluate the presence of fecal microbiota dysbiosis. A high LDA score indicates a greater contribution of the taxa to the group separation. We detected a high difference among groups (*p* < 0.01). At the genus level, mice fed HF + 10% VFP showed a significant decrease in the abundance of *Desulfovibrionaceae* and *Enterobacteriales* with respect to those fed the HF diet without phenolic compounds. The *Desulfovibrionaceae* species are known to produce significant amounts of sulfuric acid (H_2_S), which can cause oxidative stress and is correlated with inflammatory bowel disease [57]. In this way, Duda-Chodak et al. [58] reported that different polyphenols can retard the growth in vitro of potentially detrimental species, such as *Clostridiales* and *Enterobacteriales* as *Escherichia coli* or *Salmonella thiphy.* High-fat diets induce severe disruptions in colonic microbial communities. Fornelos et al. [59] observed that with the increased ratio of omega-6: omega-3 fatty acid in the diet (such as the HF diet), the symbiotic bacteria that maintain host immunological equilibrium are affected, whereas the growth of pathogenic bacteria favoring inflammatory diseases is favored. Our results showed that incorporating VFPE (0.5%, 1%, and 2%) reduced the abundance of *Erysipelotrichales*, *Chloroplast, Mollicutes,* and *Mycoplasmatales* genus of the group of Firmicutes. Interestingly, the evidence from in vitro and in vivo studies shows that a regular intake of polyphenols and their metabolites exert positive effects on colonic microbial communities, promoting gut health by shifting the gut microbial composition from dysbiosis to eubiosis via stimulating the growth of beneficial bacteria while inhibiting the invasion and establishment of pathogenic ones. The use of black and green teas, plum, peach, grape, mango peel, olive pomace, and other vegetable sources of prebiotics promotes beneficial microbial composition, increasing *Bifidobacterium* spp., *Lactobacillus* spp., and *Enterococcus* spp. All of these bacteria produce and release short-chain fatty acids (SCFAs), which exert numerous beneficial metabolic effects. Prebiotic vegetable intake also decreases the abundance of *Prevotella*, *Bacteroides*, *Clostridium hystolyticum,* and other pathogenic bacteria [60,61,62,63,64,65,66] with beneficial metabolic and immune effects in the host.

### 3.8. VFPE Reverted Visceral and Subcutaneous Adipocyte Hypertrophy and Restored Thermogenic Brown Adipose Tissue Morphology in Obese Mice

Preventive protocols allow us to evaluate the effect of a particular strategy, such as dietary manipulation, on the development of metabolic abnormalities in healthy animals exposed to an obesogenic environment. These approaches are important to gain insight into the mechanisms activated that lead to metabolic derangements and the capacity of the strategy to prevent the activation of these mechanisms. However, a more challenging approach is the therapeutic protocol, where the strategy is tested in animals with metabolic derangements. For this purpose, we fed obese mice with VFP to evaluate their therapeutic potential. We found that consuming VFP for ten weeks reverted excessive weight gain and visceral and subcutaneous adipocyte hypertrophy and restored thermogenic brown adipose tissue morphology. Unexpectedly, VFP intake did not improve glucose tolerance, indicating that VFP intake can partially improve metabolic alterations induced by chronic high-fat diet intake in mice.

## 4. Materials and Methods

### 4.1. Vegetal Collection and Pods Extract

The *Vachellia farnesiana* pods (VFP) used in this study were collected in Acatlán de Osorio in the state of Puebla in México, located between 18°0402400 and 18°2103000, north latitude and 97°5501800 and 98°1102400 west longitude; *Vachellia farnesiana* was registered with an internal identification number (8757) at the herbarium of the Facultad de Estudios Superiores Cuautitlán at the Universidad Nacional Autónoma de México (UNAM). The pods were dried in the shade at room temperature and then ground in a knife mill to obtain medium-textured flour. Additionally, the *Vachellia farnesiana* pods extract (methanol:water (80:20, *v*/*v*) or distilled water) was obtained following the instructions described by Delgadillo-Puga et al. [19].

### 4.2. Quantification of Methyl Gallate and Naringenin from VFP

To determine the quantify of MG and NA in one gram of raw methanol:water 80:20 extract of VFP, we used one gram of extract that was passed through column chromatography on silica gel (20 g) and eluted with a gradient of hexane/EtOAc. Each fraction was analyzed on TLC, and the fractions rich in MG were joined in pooled fractions. Alternatively, fractions rich in NG were joined in other pooled fractions. Both pooled fractions were analyzed separately to quantify MG and NA using high-performance liquid chromatography with a charge-coupled device detector (HPLC: SY-8100 Beijing Beifen-Ruili Analytical Instrument (Group) Co., Ltd. Beijing, China, and CCD: USB2000, Ocean Optics Inc. Orlando, FL, USA). The separation of the analytes was carried out at room temperature using a Phenomenex Luna C8 column as a stationary phase (150 mm × 4.6 mm, 5 µm). The mobile phase was acetonitrile: water acidified with 1.0 % (*v*/*v*) acetic acid (35:65, *v*/*v*) at a 1 mL/min flow rate, and the injection volume was 100 µL [33]. The detection wavelength was set at 270 nm for MG and 283 nm for NA, as previously determined by our research group.

### 4.3. In Vivo Assay

#### 4.3.1. Animals

Fifty-four male C57BL/6 mice of 5 weeks of age and weighing 21–23 g were obtained from the Experimental Research Department and Animal Care Facility at the Instituto Nacional de Ciencias Médicas y Nutrición Salvador Zubirán (DIEB-INCMNSZ) and housed in micro isolator cages at 23 °C with a 12-h on/12-h off light–dark cycle (7:00 a.m.–7:00 p.m.). All animal procedures were conducted following the recommendations and procedures from the National Institutes of Health Guide for care and use of Laboratory Animals [67]. The Animal Care Committee of the Instituto Nacional de Ciencias Médicas y Nutrición Salvador Zubirán (CINVA-INCMNSZ) approved the study (Approval number CICUAL-NAN-1988-20-22-1).

#### 4.3.2. Experimental Design and Diets

The in vivo studies in mice were divided in two experimental designs: The first was a prevention study, where mice were randomly divided in seven experimental groups (*n* = 6) receiving one of the following isoenergetic experimental diets: (1) Control; (2) High-fat (HF); (3) HF + 0.1% methyl gallate (HF 0.1 MG; Cat. 274194; Sigma Aldrich); (4) HF + 10% *Vachellia farnesiana* pods (HF 10 VFP); (5) HF + 0.5% *Vachellia farnesiana* polyphenol extract (HF 0.5 VFPE); (6) HF + 1% of *Vachellia farnesiana* polyphenol extract (HF 1 VFPE); HF + 2% of *Vachellia farnesiana* polyphenol extract (HF 2 VFPE). The diets were administered in dry form, and their composition (Table 1) was adjusted according to the recommendations of Reeves et al. [68]. This essay had a duration of fourteen weeks. The second experimental design was an intervention study, where two groups (*n* = 6) of mice were fed a high-fat (HF) diet for fourteen weeks, and at the end of this period, one group was switched to an HF + 10% of *Vachellia farnesiana* pods (HF 10 VFP) diet for ten additional weeks.

**Figure 12:**
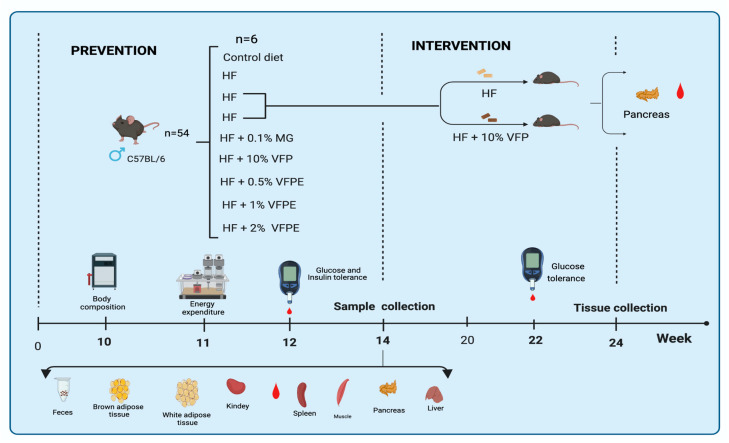
Dietary experimental design (prevention and intervention). We used male mice fed with a Control diet (Control), High-fat diet (HF), HF diet containing 0.1% methyl gallate (HF 0.1 MG), 10% *Vachellia farnesiana* pods (HF 1 VFP), 0.5% (HF 0.5 VFPE), 1% (HF 1 VFPE), or 2% (HF 2 VFPE) of *Vachellia farnesiana* pods phenolic extract.

All animals had ad libitum access to water and their respective experimental diet in both stages. Body weight was measured once a week, and food intake was measured every other day during all studies. Body composition, energy expenditure, glucose, and insulin tolerance were evaluated at weeks 10, 11, and 12, respectively, as indicated below. After eight weeks of stage II (intervention diet), glucose tolerance was evaluated for 22 weeks. The animals assigned to intervention completed 24 weeks of study (Figure 12).

The study concluded with a 6-h food deprivation for the mice before euthanasia, which was carried out by exposing them to a lethal dose of sevoflurane. To obtain the serum, a 1 mL syringe coated with heparin was used to draw the total blood volume from the posterior vena cava. The serum was then separated by centrifugation for 10 min at 1500× *g* at 4 °C. Subsequently, various organs including subcutaneous adipose tissue (SAT), visceral adipose tissue (VAT), brown adipose tissue (BAT), liver, pancreas, spleen, skeletal muscle (soleus and gastrocnemius), and kidneys were quickly removed and divided into small parts. Some of these samples were frozen in liquid nitrogen and stored at −80 °C while the remaining samples were fixed in ice-cold 4% (*w*/*v*) paraformaldehyde in phosphate buffer saline (PBS) for histological analyses, as described below.

#### 4.3.3. Evaluation of Body Composition and Energy Expenditure

Lean and fat mass were determined at week 11 using magnetic resonance imaging (EchoMRI; Echo Medical Systems, Houston, TX, USA). Energy expenditure and substrate utilization were measured at week 12 by indirect calorimetry using the Oxymax CLAMS system (Comprehensive Laboratory Animal Monitoring System, Columbus, OH, USA). Briefly, mice were acclimatized for six h in the CLAMS cages before data collection. The animals were food deprived for 8 h for fasting recordings and then fed their corresponding diets for the next 18 h. Oxygen (O_2_) consumption and carbon dioxide (CO_2_) production were continuously measured throughout the test. The respiratory exchange ratio (RER) was determined as the volume of CO_2_ exhaled (VCO_2_, mL·kg^−1^·h^−1^) divided by the volume of O_2_ used (VO_2_, mL·kg^−1^·h^−1^).

#### 4.3.4. Evaluation of Glucose and Insulin Tolerance

Intraperitoneal glucose tolerance tests (ipGTT) and insulin tolerance tests (ipITT) were performed on weeks 12 and 13, respectively. Mice were food-deprived for six hours before each test. The IpGTT was initiated by an intraperitoneal injection of 2 g/kg of glucose and the ipITT by an intraperitoneal injection of 0.5 UI/kg of insulin. Glucose was measured in blood from the tail vein at 0, 15, 30, 45, 60, 90, and 120 min after the injection using a portable FreeStyle glucometer (Abbott, Mexico City, Mexico).

#### 4.3.5. Histological Analysis of Liver, Pancreas, White and Brown Adipose Tissue

Samples of the liver, pancreas, and adipose tissues were fixed in PBS-buffered 4% paraformaldehyde, dehydrated, embedded in paraffin, and cut into 4 µm slices. Sections were stained with hematoxylin and eosin (H&E) and observed under a Leica DM750 microscope (Leica, Wetzlar, Germany). The adipocyte areas were analyzed using the Adiposoft software for ImageJ (ImageJ 1.52 K, National Institutes of Health, Bethesda, ML, USA); additionally, the same software was used to evaluate pancreatic islet areas. The perimeter of each islet was drawn manually after calibrating the software using the 100 µm scale bar. The number of islets was measured in two sections of each pancreas at different depths. Digital photographs were taken from each section at 20× magnification.

#### 4.3.6. Mitochondria Abundance in Skeletal Muscle

To assess the abundance of mitochondria and lipid content in skeletal muscle, the gastrocnemius and soleus muscles were frozen in optimal cutting temperature compound (OCT) and stored at −80 °C. Succinate dehydrogenase (SDH) activity was used to visualize the mitochondrial abundance, as previously reported [20]. Frozen sections of 12 μm were mounted onto positively charged slides and incubated with SDH staining solution containing nitro-blue tetrazolium and sodium succinate at 37 °C for 60 min. The slides were washed with deionized water and dehydrated in 30%, 60%, and 90% acetone sequentially for 2 min each, then rehydrated with 60% and 30% acetone in deionized water for another 2 min each. Digital photographs were captured at 20× magnification, and positive fibers (appearing blue) were quantified using Image J 7 software, as previously described [14].

#### 4.3.7. Lipid Content in Liver and Skeletal Muscle

To visualize neutral lipid accumulation in metabolic tissues, frozen liver and skeletal muscle samples were sectioned with a cryostat (8 μm) and stained with 0.5% Oil Red O (ORO) in propylene glycol (Sigma-Aldrich, St. Louis, MO, USA). For the quantitative analysis of the ORO staining, images were converted to an 8-bit grayscale in ImageJ 7 software (National Institutes of Health, Bethesda, MD, USA; https://imagej.nih.gov/ij/ (accessed on 28 February 2023) according to Mehlem et al. [69] to measure the area and mean gray value, which is multiplied to obtain the integrated density value.

#### 4.3.8. Kidney Histological Analysis

At the end of the experiment, the kidneys were rapidly removed, sliced longitudinally, and immersed in ice-cold 10% paraformaldehyde in a phosphate-buffered saline solution. After dehydration, kidney slices were embedded in paraffin, sectioned at 4 µm, and stained using hematoxylin and eosin (H&E) and trichromic-Masson methods. A computer-assisted color image analyzer (Q/win-500, Leica, Milton Keynes, Cambridge, UK) was employed to study 20 randomly selected glomeruli from each animal at ×400 magnification. Glomerular size and urinary space were measured in tissue sections stained with H&E. The percentage of glomerular surface area occupied by mesangial cellularity and extracellular matrix was obtained by normalizing the area of the trichromic-Masson-positive material in each glomerulus. Glomerular sections were displayed on the computer screen, and their area was measured by an interactive procedure with an image analysis software package. To normalize the structure for measuring, only glomeruli in which both poles: vascular and tubular, were seen were considered to perform these determinations.

#### 4.3.9. Protein Extraction, SDS/PAGE, and Immunoblotting

Tissues were homogenized in ice-cold RIPA buffer (phosphate-buffered saline (PBS), 1% IGEPAL, 0.5% sodium deoxycholate, 0.1% sodium dodecyl sulfate, 1 mM sodium fluoride, 2 mM sodium orthovanadate) containing a protease inhibitor cocktail (Complete Mini, Roche Diagnostics, South San Francisco, CA, USA) in a TissueLyser (Qiagen, Germantown, MD, USA). The samples were incubated on ice for 30 min, centrifuged at 17,400× *g* for 15 min at 4 °C, and the supernatant was transferred to a new tube. The protein content was quantified using the Folin reagent (DC protein assay kit, Bio-Rad, Hercules, CA, USA). The protein samples (40 µg) were separated on 10% SDS-polyacrylamide gels and transferred to polyvinylidene difluoride (PVDF) membranes (0.45 µm) (Hybond-P, Amersham, GE Healthcare, Chicago, IL, USA) using a wet electroblotting System (Bio-Rad, Hercules, CA, USA). The membranes were blocked for one h with 5% non-fat dry milk and incubated with primary antibodies diluted in a blocking solution overnight. The primary antibodies were as follows: AMPKa (Santa Cruz Biotechnology, Dallas, TX, USA, Cat. sc-25792, dilution 1:1250 skeletal muscle), p-AMPK (Santa Cruz Biotechnology, Dallas, TX, USA, Cat. sc-25792, dilution 1:1250 skeletal muscle), Th2-172 (Santa Cruz Biotechnology, Dallas, TX, USA, Cat. sc-33524, dilution 1:600 skeletal muscle), GLUT 4 (Santa Cruz Biotechnology, Dallas, TX, USA, Cat. sc-53566, dilution 1:200), GAPDH (Abcam, Cambridge, UK, Cat. ab181802 dilution 1:50,000 skeletal muscle), rabbit monoclonal anti-mouse UCP-1 (Abcam, Cambridge, MA, USA, Cat. ab155117 dilution 1:1000), TNF-alpha (Abcam, Cambridge, UK Cat. ab205587, dilution 1:2000 kidney tissue), GRP78 (GeneTex Inc., Irvine, CA, USA, Cat. GTX113340, dilution 1:5000), ß-actin (Aviva System Biology, San Diego, CA, USA, Cat. OAPB00400 dilution 1:2000). Membranes were washed three times with TBS-t for 10 min and then incubated with horseradish peroxidase-conjugated secondary antibodies (goat anti-rabbit or rabbit anti-goat 1:50,000 and 1:10,000) for 1 h. Immunoblots were developed using a chemiluminescent Western blotting kit (Immobilon Western Chemiluminescent HRP Substrate, Millipore, Burlington, MA, USA) and ChemiDoc MP densitometer and analyzed using Image Lab 6.1 software (Bio-Rad, Hercules, CA, USA). The results are reported as an interest protein/housekeeping protein ratio or phosphorylated/total protein ratio for phosphorylated proteins. A value of 1 was arbitrarily assigned to the Control group, which was used as a reference for the other conditions. The results are reported relative to ß-actin to the kidney and GAPDH (Abcam, Cambridge, MA, USA, Cat. ab8245) to the rest of the tissues.

#### 4.3.10. Mononuclear Cell Preparations and Flow Cytometric Analysis

Spleens were isolated from mice. Splenocytes were obtained by perfusion with PBS, and erythrocytes were lysed with ACK lysing buffer (Fisher Scientific Company, Ottowa, Ontario, Can, Cat. A10492-01) with 1 mL for 1 min and then washed with 10 mL of PBS. Samples were centrifuged at 250× *g* for 10 min at 4 °C. Then, 1–2 × 10^6^ cells/mL of RPMI 1640 (Gibco, Fisher Scientific, Madrid, España) were stimulated with 1 mL of cell activation cocktail with Brefeldin A (phorbol-12-myristate 13-acetate 40.5 mM BioLegend, Cat 423303) for 6 h. Cells were washed and resuspended with 1 mL of cold BD Cytofix^TM^ buffer (Cat. 554656) and incubated for 20 min at room temperature. The cell suspension was washed with a Perm/Wash^TM^ buffer and incubated at room temperature for 15 min. Cells were centrifuged at 250× *g* for 10 min at room temperature. The pellet was resuspended in 50 mL of Per/Wash^TM^ and added with 20 mL/tube of cocktail (CD4 PERCP-CY5.5, IL-17A PE, IFN-g FITC, and IL-4 APC) (mouse Th1/Th2/Th17 phenotyping kit Cat. 560758) and another tube with 5 μL of anti-CD4-FITC-labelled monoclonal antibody (Becton Dickinson, Biosciences, San Jose, CA, USA) and PE-labelled anti-Foxp3 for Tregs (Becton Dickinson, Biosciences, San Jose, CA, USA) at room temperature in the dark for 20 min. After two washes, cells with Perm/Wash solution splenocytes were analyzed by flow cytometry with an Accuri C6 (Becton Dickinson, Biosciences, San Jose, CA, USA). To avoid false positive PE results and set compensation for multicolor flow cytometric analysis, we performed instrument calibration/standardization procedures each day according to established protocols of our laboratory. We ran an unstained (autofluorescence control) and permeabilized splenocytes sample. Autofluorescence control (unstained cells) was compared with single-stained cell-positive Controls to confirm that the stained cells were on the scale for each parameter. Additionally, Becton Dickinson Calibrite 3 beads were used to adjust instrument settings, set fluorescence compensation, and check instrument sensitivity (CaliBRITE, Becton Dickinson, Biosciences, San Jose, CA, USA). An electronic gate was made for CD4^+^ cells. Results are expressed as the relative percentage of IL-17A^+^, IL-4^+^, IFN-γ^+^, and Foxp3^+^ expressing cells in each gate. As isotype Control, IgG1-FITC/IgG1-PE/CD45-PeCy5 mouse IgG1 kappa (BD Tritest, Becton Dickinson, Biosciences, San Jose, CA, USA) was used to set the threshold and gates in the cytometer. Fluorescence minus one (FMO) Controls were stained in parallel using the panel of antibodies with the sequential omission of one antibody, except for the anti-IL-17A, anti-IL-4, anti-IFN-γ, anti-Foxp3 antibody, which was replaced by an isotype control, (rather than Becton Dickinson, Biosciences, San Jose, CA, USA). Each sample recorded one hundred thousand events and was analyzed with the FlowJo v10.8 software (Tree Star, Inc., Ashland, OR, USA).

#### 4.3.11. Fecal DNA Extraction and 16S rRNA Sequencing

A fecal sample was collected from all animals after 14 weeks of the dietary treatment with *Vachellia farnesiana* or its extracts. Fecal samples were frozen at −80 °C. The isolation of DNA was performed using a QIAamp DNA Stool Mini Kit (Qiagen, Germantown, MA, USA), according to the manufacturer’s instructions. The variable regions 3–4 of the 16S rRNA gene were amplified using specific forward (5″ TCGTCGGCAGCGTCAGATGTGTATAAGAGACAGCCTACGGGNGGCWGCAG 3″) and reverse primers (5″ GTCTCGTGGGCTCGGAGATGTGTATAAGAGACAGGACTACHVGGGTATCTAATCC 3″) containing the Illumina adapter overhang nucleotide sequences. Ampure XP bits were used to purify the 16S V3-V4 amplicons and were quantified on TapeSation (Agilent, Santa Clara, CA, USA). The size of the amplicons was approximately 550 bp. An index PCR was then carried out to attach dual indices using a Nextera XT V2 Kit. The amplicon size was approximately 610 bp, and the concentration of double-stranded DNA was measured using a fluorometer Qubit 3.0 (Invitrogen, Waltham, MA, USA) with a high-sensitivity kit. The final amplicon library was pooled in equimolar concentrations. Sequencing was performed on the Illumina NovaSeq platform (Illumina, San Diego, CA, USA) to generate paired-end reads of 250 bases in length in each direction.

#### 4.3.12. Sequence Analysis

Illumina fastq reads were processed using the Quantitative Insights Into Microbial Ecology 2 (QIIME 2) software package. Denoising quality, chimera check, and clustering were performed using the dada2 denoise-paired instruction. The SILVA database (release_138) was used as the reference 16S rRNA database, together with the naïve Bayes algorithm-based trained classifier for a taxonomic assignment at 97%, using the feature-classifier classify-sklearn instruction. A rooted phylogenetic tree was generated for further statistical α diversity tests and weighted and unweighted UniFrac β diversity tests and derived (Principal Coordinate Analysis) PCoA using Phyloseq17 and Vegan18 R Packages. The web-based tool Microbiome Analyst (http://www.microbiomeanalyst.ca/ accessed on 24 February 2023) was used to perform a linear discriminant analysis effect size (LEfSe) method for the assessment of microbial communities’ differences (LDA < 2).

### 4.4. In Vitro Assays

#### 4.4.1. Extracellular Flux Analysis in Primary Hepatocytes and C2C12 Myotubes

We followed the method of Berry and Friend [70] to obtain primary hepatocytes by in situ perfusions of the liver, which consists of cannulating and exsanguinating the liver in vivo, followed by continuous perfusion with collagenase. The livers were then isolated and placed in Hank’s balanced salt solution (HBSS) to disaggregate the tissue. The cell suspension was then filtered through a 70 μm mesh. The filtered hepatocytes were washed twice with HBSS and, finally, re-suspended in M199 medium supplemented with 10% fetal bovine serum (FBS), antibiotic, 0.1% bovine serum albumin (BSA), 1 nM insulin, 100 nM dexamethasone, and 100 nM triiodothyronine (T3). The cells were then seeded into an XFe96 microplate at 4000 cells/well density. The medium was changed after four hours to remove unattached cells and then incubated with Methyl gallate (MG) or Naringenin (NA) at 0, 5, 10, 20, or 40 μM for 18 h. C2C12 mouse myoblasts were cultured, proliferated in DMEM as previously reported [71], and differentiated on XF96 plates to obtain C2C12 myotubes. At day 6 of differentiation, C2C12 myotubes were treated as indicated for primary hepatocytes. The mitochondrial function of primary hepatocytes or C2C12 myotubes was evaluated using a mitochondrial stress test in an XFe96 Extracellular Flux Analyzer (Agilent Technologies, Santa Clara, CA, USA). Briefly, the cells were washed and incubated for 1 h in a non-CO_2_ incubator with XF basal medium supplemented with 10 mM glucose, 1 mM pyruvate, and 2 mM glutamine. During the experiment, 2 μM oligomycin, 1 μM (primary hepatocytes), or 0.5 μM (C2C12 myotubes) carbonyl cyanide-p-trifluoromethoxyphenyl-hydrazone (FCCP) and 1 μM rotenone/antimycin A were injected sequentially, and three measurements were performed in basal conditions and after the addition of each compound. The oxygen consumption ratio (OCR) measurements were obtained and analyzed according to the manufacturer’s recommendations.

#### 4.4.2. Evaluation of Insulin Secretion in INS-1E Cells

The rat insulinoma INS-1E was kindly donated by Profs. C.B. Wollheim and Pierre Maechler of the University of Geneva (Switzerland). INS-1E cells were cultured in RPMI 1640 medium supplemented with 1 mM sodium pyruvate, 2 mM glutamine 50 μM 2-mercaptoethanol, 10 mM HEPES, 10% FBS, 100 U/mL penicillin, and 100 μg/mL streptomycin under standard incubator conditions. Insulin secretion was evaluated using INS-1E cells, and the cells were seeded on 24-well plates and, after 48 h, treated with 0, 5, or 10 mg/mL of a polyphenolic extract of VFP for 2 h. Following treatment, INS-1E cells were washed 3 times with KRBB containing 2.5 mM glucose. INS-1E cells were incubated for 30 min in KRBB, including 2.5 mM glucose, then maintained at 2.5 mM glucose or switched to 8.3 mM glucose for an additional 2 h. After incubation, the medium was collected and frozen to determine insulin concentration using an ELISA kit (Merck, Lowe, NJ, USA, Cat. 41116126). Total insulin content was determined in an aliquot obtained by adding acidified ethanol (75% ethanol/1.5% HCl) to the attached cells. Insulin secretion was expressed as the percentage of secreted insulin into the media to the sum of secreted and total insulin content of experiments performed on three different cell passages.

### 4.5. Statistical Analyses

Data are expressed as mean ± standard error of the means (S.E.M.). The Shapiro–Wilk normality test was used to check data distribution. All groups were analyzed by one-way ANOVA followed by Tukey multiple comparisons post hoc tests using GraphPad Prism 7.0 (GraphPad Software, San Diego, CA, USA). The differences were considered statistically significant at *p* < 0.05. Mean values with different lowercase letters show statistical differences between each other. Additionally, the data for the intervention assay were analyzed by unpaired two-tailed *t*-test using GraphPad Prism 7.0 Software. The differences were considered statistically significant at *p* < 0.05.

## 5. Conclusions

In conclusion, the present study demonstrates that bioactive compounds in *Vachellia farnesiana* pods prevented systemic inflammation, insulin resistance, and hepatic and renal damage associated with increased gut microbiota diversity, along with increased mitochondrial function and energy expenditure. This work paves new avenues for the prevention and treatment of metabolic diseases based on dietary strategies that include non-conventional vegetable sources of phenolic compounds, such as *Vachellia farnesiana*.

## Figures and Tables

**Table 1:** Composition of experimental diets.

	g/kg of Diet						
Ingredient (%)	Control	HF	HF + 0.1% Methyl Gallate	HF + 10% VFP	HF + 0.5% VFPE	HF + 1% VFPE	HF + 2% VFPE
Casein ^a^	200	200	200	190	200	200	200
Sucrose ^b^	100	339	338	271	334	329	319
Maltodextrin ^d^	132	150	150	150	150	150	150
Cornstarch ^c^	397	0	0	0	0	0	0
Lard	0	140	140	128	140	140	140
Soy oil ^e^	70	70	70	70	70	70	70
Cellulose ^f^	50	50	50	39	50	50	50
Vitamin Mix ^g^	10	10	10	10	10	10	10
Mineral Mix ^h^	35	35	35	35	35	35	35
L-Cystine ^i^	3	3	3	3	3	3	3
Choline ^j^	2.5	2.5	2.5	3	2.5	2.5	2.5
Experimental Compounds	0	0	1	100	5	10	20
	g/100 g of diet						
Protein	20.3	17.2	17.2	18.0	17.3	17.4	17.5
Carbohydrates	63.8	42.1	42.1	40.0	41.9	41.6	41.1
Fat	16.0	40.7	40.7	42.1	40.9	41.0	41.4
	kcal/kg of diet						
Energy content	3946	4646	4642	4228	4626	4606	4566
	mg GAE/kg of diet						
Total polyphenols content	-	-	-	399.0	199.5	399.0	798.0

High-fat (HF); HF + 0.1% methyl gallate (HF 0.1 MG); HF + 10% *Vachellia farnesiana* (VF) pods dry (HF 10 VFP); HF + 0.5% of VF polyphenolic extract (PE) (HF 0.5 VFPE); HF + 1% of VFPE (HF 1 VFPE) and HF + 2% of VFPE (HF 2 VFPE). ^a^ Casein, vitamin-free test (Envigo, Teklad, Indianapolis, IN, USA). ^b^ Sucrose (cane sugar). ^c^ Cornstarch (CP ingredients). ^d^ Maltodextrin (MP Biomedicals, Irvine, CA, USA). ^e^ Soy oil (commercial oil). ^f^ Cellulose (AIN Alphacel Non-Nutritive Bulk, MP Biomedicals). ^g^ Vitamin Mix (AIN-76 Vitamin Mix, MP Biomedicals). ^h^ Mineral Mix (AIN-76 Minerals Mix, MP Biomedicals). ^i^ L-Cysteine (Sigma-Aldrich, St. Louis, MO, USA). ^j^ Choline citrate (Sigma-Aldrich). GAE = gallic acid equivalents.

## Data Availability

Not applicable.

## Supplementary Materials

The supporting information can be downloaded at: https://www.mdpi.com/article/10.3390/ijms24097984/s1.
