# Supplementary figures and images for: Vachellia farnesiana Pods or a Polyphenolic Extract Derived from Them Exert Immunomodulatory, Metabolic, Renoprotective, and Prebiotic Effects in Mice Fed a High-Fat Diet
Source: Int J Mol Sci. 2023 Apr 28;24(9):7984. doi: 10.3390/ijms24097984 (PMC10178983; doi:10.3390/ijms24097984)

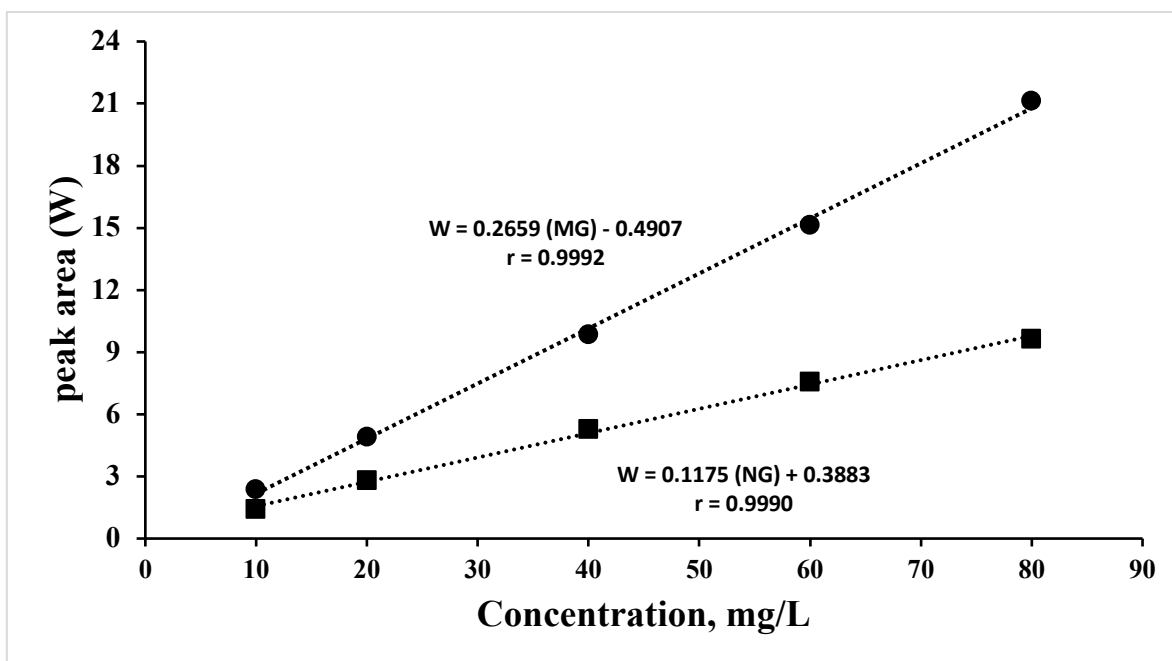

Figure S1. Calibration curve of methyl gallate (MG) ● and naringenin (NA) ■

Supplement: Supplementary file 1 [file ijms-24-07984-s001.zip › ijms-2301820-supplementary.pdf]
